# Deep-Sea Biomimetic Manta Ray Robots: A Comprehensive Review Based on Operational Depth Spectrum, Structures, Energy Optimization, and Control Systems

**DOI:** 10.3390/biomimetics11030216

**Published:** 2026-03-18

**Authors:** Lugang Ye, Hongyuan Liu, Qiulin Ding, Zhongming Hu, Weikun Li, Weicheng Cui, Dixia Fan

**Affiliations:** 1 Department of Electronic and Information Engineering, School of Engineering, Westlake University, Hangzhou 310030, China; yelugang@westlake.edu.cn (L.Y.); liuhongyuan@westlake.edu.cn (H.L.); dingqiulin@westlake.edu.cn (Q.D.); cuiweicheng@westlake.edu.cn (W.C.); 2 College of Building Eneineering, Huanghuai University, Zhumadian 463000, China; hu-zhongming@huanghuai.edu.cn; 3 Zhejiang Engineering Research Center of Micro/Nano-Photonic/Electronic System Integration, Hangzhou 310030, China

**Keywords:** bio-inspired submersible, manta ray, biomimetic propulsion, full-ocean-depth

## Abstract

As deep-sea exploration transitions from large-scale search to precision pinpoint operations, the inherent limitations of traditional “rigid-body and propeller” vehicles—specifically in low-speed maneuverability, environmental compliance, and acoustic stealth—are becoming increasingly apparent. Leveraging its unique integrated “gliding-flapping” locomotion and exceptional maneuverability, the manta ray serves as an ideal biological prototype for next-generation deep-sea operational platforms. From a systems engineering perspective, this paper provides a comprehensive review of the current research status and technical evolution of biomimetic manta ray submersibles. First, a technical pedigree centered on “operational depth” is established, illustrating how design paradigms transition from “mechanism replication” in shallow waters to “pressure adaptation” at full-ocean depths. Second, the mechanical challenges in structural design are explored, demonstrating that a “rigid-flexible” gradient distribution strategy is critical to resolving the conflict between pressure resistance and propulsive compliance. Regarding energy and propulsion, the synergistic effects of hybrid gliding-flapping drives and integrated structural batteries in enhancing long-range endurance and energy efficiency are analyzed. Finally, the evolution of motion control architectures—transitioning from spinal-cord-inspired Central Pattern Generator (CPG) rhythmic control to Deep Reinforcement Learning (DRL) featuring embodied intelligence—is outlined.

## 1. Introduction

### 1.1. Structural Limitations of Conventional Underwater Vehicles

The ocean, covering 71% of the Earth’s surface, represents the final frontier for resource acquisition and national security strategies [1,2]. As human exploration extends from the shallow continental shelves to the deep-sea hadal zones, the mission profile of underwater robots is undergoing a fundamental shift: from traditional wide-area seafloor mapping and reconnaissance based on acoustic payloads toward fine-scale verification of high-value targets, in situ biological sampling, deep-sea facility maintenance, and cross-medium docking operations [3,4]. As noted by Wang these unstructured and complex environments pose unprecedented challenges to the configurations of underwater robots, as traditional single-rigid-body designs struggle to meet the stringent environmental adaptability requirements of future underwater platforms [5,6,7].

Against this backdrop of evolving mission requirements, the “rigid body of revolution plus screw propeller” design paradigm that has long dominated the subsea engineering field is increasingly revealing its limitations [8]. Although conventional torpedo-shaped Autonomous Underwater Vehicles (AUVs) have reached technical maturity in low-drag, constant-speed cruising, they face irreconcilable contradictions during close-range operations in complex deep-sea terrains, such as hydrothermal vents and submarine canyons [9]. Research by Wright, combining numerical simulations and experimental validation, demonstrated that biomimetic propulsion systems exhibit superior controllability in complex terrains compared to traditional rigid structures [10].

First, propulsion methods based on rotary blades suffer from a sharp attenuation of control authority at low or zero speeds, making it difficult to achieve precise six-degree-of-freedom (6-DOF) hovering and attitude adjustment. In an analysis of hydrodynamic performance in high-Reynolds-number environments, Konstantinos further pointed out that traditional BCF (Body and/or Caudal Fin) and propeller-driven methods encounter efficiency bottlenecks under the influence of deep-sea high pressure and high viscosity; thus, there is an urgent engineering need to shift toward flexible propulsion to enhance adaptability and maneuverability [11]. Roper also reviewed the application potential of biological propulsion systems in AUVs [12], concluding that biomimetic propulsion offers significant advantages in low-speed maneuvering and acoustic stealth [13]. Second, rigid buoyancy regulation and attitude control systems lack the flexible compliance necessary to handle complex deep-sea internal waves and turbulence. Consequently, robots must frequently execute corrections to maintain their attitude, leading to energy spikes and reduced endurance [14]. Furthermore, the strong shear flows and cavitation noise generated by high-speed rotating propeller blades not only disturb deep-sea organisms—thereby undermining the authenticity of in situ ecological observations—but also lack the acoustic and physical stealth required for operations in high-risk areas [15].

**Figure 1:**
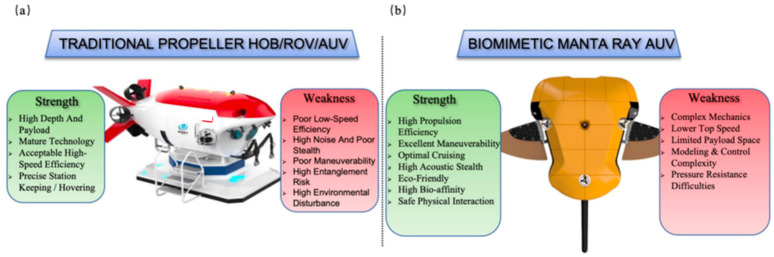
Comparison of technical characteristics between traditional propeller-driven submersibles and biomimetic manta ray submersibles.(**a**) Traditional propeller HOB/ROV/AUV; (**b**) Biomimetic manta ray AUV.

As shown in Figure 1, In response to the asymptotic effect of traditional engineering technologies, scientists have turned their attention to marine organisms shaped by hundreds of millions of years of natural selection [16,17]. By summarizing cross-species locomotor mechanisms, Liu demonstrated the systematic guiding significance of biological strategies for engineering fluid design [18]. Paig-Tran, starting from shark skeletal structures, emphasized that biomimetic systems need to reconstruct the matching mechanism between structure and performance, providing a biological rationale for the paradigm shift in deep-sea robot design [19]. Salazar further clarified the advantages and disadvantages of various biological prototypes in engineering implementation through classification studies of biological and biomimetic underwater systems [20]. Evolutionary theory indicates that marine organisms have achieved a perfect unification of propulsive efficiency, maneuverability, and environmental adaptability through the long-term co-evolution of skeletons, muscles, and the fluid environment. Among these, the manta ray stands out as an exceptional representative of cartilaginous fish, distinguished by its unique “integrated glide-and-flap” locomotor mode [21].

### 1.2. Bio-Fluid Mechanic Mechanisms of Manta Ray Swimming

**Figure 2:**
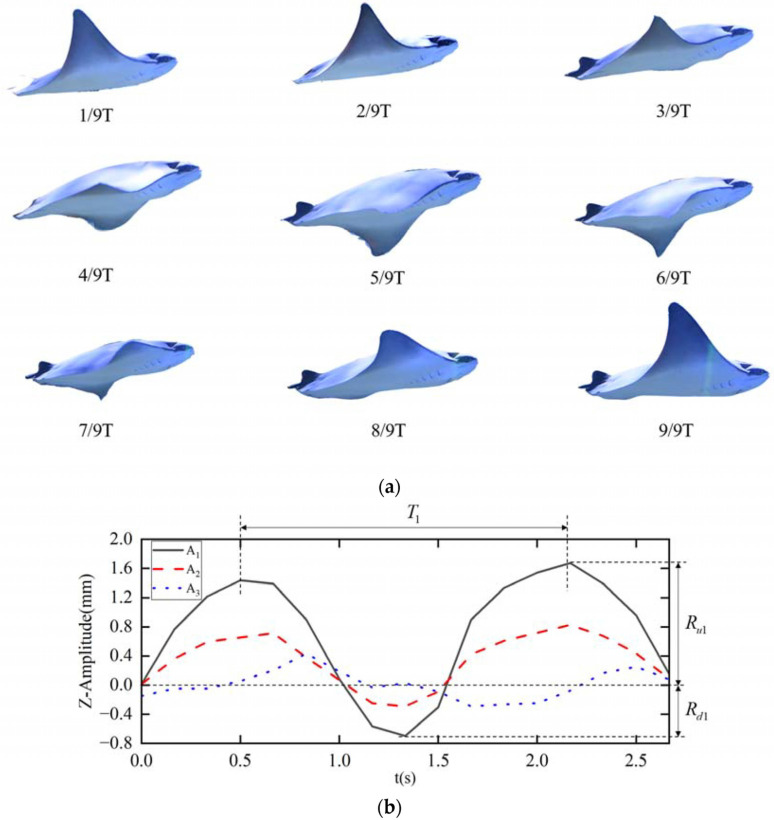
Technical lineages and evolutionary logic of biomimetic manta ray submersibles based on operational depth, (**a**) chordal motion sequence of the pectoral fin; (**b**) motion trajectory of manta ray feature points.Adapted from Ref. [28], used under CC BY 4.0.

Across the physical environmental gradients ranging from shallow waters to full-ocean depths, the performance evolution of the traditional “rigid revolution body plus propeller” configuration differs distinctively from that of the biomimetic “flexible flapping wing” propulsion, As shown in Figure 2.

First, regarding propulsion efficiency, traditional propellers face severe challenges from “high pressure-viscosity” effects as depth increases. Konstantinos’s analysis indicates that in deep-sea environments characterized by high pressure and high viscosity, traditional BCF (Body/Caudal Fin) and propeller-driven modes encounter significant efficiency bottlenecks, leading to a sharp decline in energy conversion rates [11]. More critically, to withstand the extreme hydrostatic pressure of the deep sea, traditional AUVs must employ thick-walled metal pressure hulls, resulting in a “weight-buoyancy vicious cycle” [22]. The “dead weight” effect of this rigid structure severely compresses the battery payload space, causing the system-level energy density of rigid-hull AUVs to be far lower than that of individual battery cells. In contrast, biomimetic manta rays utilize “flexible deformation” to comply with rather than resist the fluid. Experimental observations demonstrate that biological manta rays achieve peak propulsion efficiencies of 89–92% during cruising [15]. Their flapping frequency adheres to the Strouhal number law [23,24]; when the St value is maintained within the “sweet spot” of 0.2–0.4 [25], flexible flapping wings generate maximum thrust coefficients with minimal energy dissipation [26], thereby demonstrating a Cost of Transport (CoT) far superior to that of propellers during long-distance deep-sea migration.

Second, in the dimension of maneuverability, there is a fundamental difference in control authority within the low-speed regime. Traditional propulsion based on rotating blades suffers a sharp reduction in control surface effectiveness (rudder effect) and control moments at low or zero speeds, making it difficult to achieve the six-degree-of-freedom (6-DOF) hovering and fine attitude adjustments required for complex deep-sea terrains, such as hydrothermal vents and submarine canyons. Conversely, biomimetic manta ray robots utilize asymmetrical flapping or “anti-phase” control of the pectoral fins to leverage the difference in fluid form drag, enabling zero-radius turning and rapid braking maneuvers. Wright’s research confirms via numerical simulation that this biomimetic propulsion system, based on unsteady hydrodynamics, exhibits maneuverability unmatched by traditional rigid structures when dealing with deep-sea internal wave disturbances and complex terrain avoidance [10].

Finally, concerning high-pressure adaptability and acoustic stealth, the high-speed rotation of traditional propellers generates strong shear flows and cavitation noise, which disrupts the authenticity of in situ ecological observations. Furthermore, their rigid transmission mechanisms are prone to stress concentration and fatigue fracture under deep-sea high pressure. In contrast, biomimetic propulsion adopts a “rigid-flexible coupling” structure [17], utilizing the passive deformation of flexible materials to delay the shedding of the Leading Edge Vortex (LEV) [27]. This mechanism not only maintains hydrodynamic stability in high-pressure environments but also inherently possesses low-noise characteristics, significantly enhancing the stealth and survivability of deep-sea operations.

The locomotor mode of the manta ray is a typical oscillatory mode of Median and Paired Fin (MPF) propulsion [29,30]. Unlike fish that rely on BCF undulations, the manta ray primarily utilizes a pair of massive triangular pectoral fins for large-amplitude up-and-down flapping. This motion is essentially a lift-based propulsion method; when the pectoral fins flap in the fluid, they act as a pair of high-aspect-ratio flexible wings performing unsteady motion [31]. In reviewing the transition path of biomimetic propulsion, Wang, W underscored that such unsteady aerodynamic mechanisms are the core for translating natural swimming into engineering robotic systems [32].

The key to its highly efficient propulsion lies in the generation and control of the Leading-Edge Vortex (LEV) [33,34]. Menzer, Zhang and Hirai systematically elucidated the formation and stabilization mechanisms of the LEV, pointing out its critical role in biological flight and biomimetic propulsion, which constitutes the theoretical foundation of biomimetic fluid dynamic design [35,36,37,38]. During the downstroke, the fluid separates as it flows over the leading edge of the pectoral fin, forming a low-pressure spiral vortex tube attached to the upper surface of the wing—the LEV [27]. While traditional fixed-wing aviation theory suggests that high angles of attack lead to flow separation and subsequent stall, the spanwise flexibility and chordwise deformation of the manta ray’s pectoral fins effectively stabilize the LEV, preventing its premature shedding. This stable low-pressure vortex provides substantial suction to the pectoral fin, generating significant lift and thrust [39]. Through numerical simulations, Zhang further revealed the vortex dynamic mechanisms in the oscillatory swimming of manta ray pectoral fins [40], finding that at high Strouhal numbers, the shedding of tip vortices is the primary cause of thrust generation [41,42]. Comparative analyses of active and passive flexible structures by Nayak also confirmed that active flexible deformation can significantly enhance propulsive efficiency and adaptability in complex flow fields [43], further validating the advantages of the manta ray’s active pectoral fin deformation.

Experimental observation data indicate that the peak propulsive efficiency of biological manta rays in a cruising state can reach 89–92% [15], exceeding the performance of traditional propellers at equivalent Reynolds numbers. The manta ray-inspired soft robot developed by Katzschmann represents a milestone in verifying this mechanism; its “integrated glide-and-flap” mode demonstrated low-speed control and energy efficiency superior to traditional rigid propulsion in experiments [44].

The hydrodynamic advantages of manta ray swimming are also reflected in the optimal matching of its dynamic parameters. The relationship between flapping frequency, flapping amplitude, and cruise velocity follows the Strouhal number law [23,24]. Classic research by Triantafyllou pointed out that when is within the 0.25–0.35 range, the efficiency of flexible unsteady propulsion far surpasses that of traditional propellers [45]. This theoretical value is strikingly consistent with biological measurement data—observations by Fish showed that the value of manta rays during long-distance migration is consistently maintained within the 0.2–0.4 range [46]. This interval has been confirmed by hydrodynamic stability analysis as the “sweet spot” [25], where the vortex street can generate the maximum thrust coefficient with minimum energy dissipation [26].

However, although the biological mechanisms are relatively clear, the process of translating them into engineering equipment is not a simple biomimetic replication; a significant performance gap remains between the two. Most current biomimetic robotic fish can only verify mechanisms in low-Reynolds-number shallow-water environments, facing great difficulty in deep-sea engineering applications. This disparity primarily stems from the property mismatch between engineering materials and biological tissues: synthetic engineering materials currently struggle to replicate the sophisticated non-uniform, variable-stiffness characteristics of biological muscle-skeletal systems [47]. In a review, Ma explicitly pointed out that while smart materials such as shape memory alloys and piezoelectric materials have been applied to robotic fish, physical limitations in material performance remain the primary bottleneck hindering the efficient engineering translation of current biomimetic propulsion [48]. Furthermore, the power density and energy conversion efficiency of existing drive mechanisms further attenuate in high-pressure deep-sea environments, resulting in a system-level Cost of Transport (COT) that is often an order of magnitude higher than that of biological counterparts. Addressing these engineering gaps is a pressing issue for current research.

### 1.3. Core Scientific Challenges in Deep-Sea Biomimetic Propulsion

While the biological mechanisms of the manta ray provide an ideal theoretical model, translating these biological discoveries into deep-sea engineering equipment still faces a massive scientific divide. Wang, X and Wood pointed out that this challenge is inherently interdisciplinary, requiring the collaborative resolution of deep coupling issues across fluid mechanics, materials science, energy systems, and control theory [49].

First, the mechanical paradox between structural pressure resistance and flexible propulsion. Biological pectoral fins are flexible, inducing the generation of LEVs to produce thrust through active deformation [17]. However, the traditional logic of deep-sea engineering requires structures to possess extremely high rigidity to resist hydrostatic pressure [22]. Maintaining the flexibility required for hydrodynamics under high pressure while avoiding structural buckling failure is a core contradiction in current structural design. To address this, Lu proposed that “rigid-flexible coupled” variable-stiffness structures are a key evolutionary direction for handling the conflict between deep-sea high pressure and flexible propulsion [50], the biomimetic soft robot design methodology proposed by Li also validated that such hybrid structures possess higher robustness in irregular sea conditions [51].

Second, the metabolic contradiction between limited energy and infinite endurance. Deep-sea missions often require robots to possess self-sustained capabilities for weeks or even months. With battery energy density limited by electrochemical physical boundaries, simply increasing battery capacity leads to a “weight-energy” death spiral. Borrowing from biological energy utilization mechanisms—such as utilizing ocean current gliding, Ocean Thermal Energy Conversion (OTEC), or integrated structural-battery designs—to construct an efficient “robotic metabolic system” is the key to achieving long endurance [52]. Weihs hypothesized as early as 1973 that fish schools utilize flow fields to save energy [53], and modern research has further confirmed the feasibility of using vortex fields for energy conservation [54].

Third, the cognitive gap between model uncertainty and precision control. Unlike rigid-body robots, the biomimetic manta ray is an infinite-dimensional flexible continuum system. Its fluid-structure interaction (FSI) effects in unsteady flow fields are extremely complex, making it difficult to establish precise analytical models. Zhang emphasized that Computational Fluid Dynamics (CFD) analysis of FSI is crucial for understanding and optimizing biomimetic propulsion performance in complex deep-sea environments and is a necessary means to break the limitations of analytical frameworks [55]. Traditional Proportional-Integral-Derivative (PID) control often fails when facing such highly nonlinear systems. To address this challenge, Xu N W, Wang X, and Shahid F proposed a soft robot scheme with vortex-sensing capabilities, integrating biomimetic skin to cope with turbulence and internal waves, thereby providing a new perceptual dimension for attitude stability control in deep-sea operations [49,56,57]. Consequently, combining CPG with DRL to achieve active adaptation to complex flow fields is the necessary path toward endowing robots with “embodied intelligence” [58].

### 1.4. Review of Related Work

In recent years, with the burgeoning development of biomimetic underwater vehicle technology, the academic community has extensively reviewed its progress from multiple dimensions. Regarding fundamental classification and actuation mechanisms, Chu (2012) [59] proposed a framework based on swimming modes and actuator types, examining how smart actuators enhance the thrust-to-weight ratio of microrobots. Addressing the evolution of actuation materials, Ma (2023) [48] provided an in-depth analysis of smart materials—such as IPMCs, SMAs, and EAPs—in micro and soft robotic fish, emphasizing their pivotal role in achieving compact and silent submersibles. Meanwhile, Claudio (2021) [60] offered a detailed comparison between rigid crank-slider mechanisms and flexible smart materials for reproducing pectoral fin motions. As deep-sea exploration demands increase, the environmental adaptability of soft robots has garnered significant attention. Bu (2022) [61] analyzed fluidically actuated (hydraulic/pneumatic) soft robots, highlighting their superior compressive resilience in high-pressure environments, while Plum (2020) [62] verified the effectiveness of compliant exoskeletons in absorbing collision energy to protect internal electronics. Furthermore, early research by Katzschmann (2018) [44] demonstrated that fully soft robotic fish can operate alongside marine organisms without disturbance, opening new avenues for behavioral ecology research.

In the field of hydrodynamics and propulsion strategies, Li (2023) [63] categorized undulatory propulsion into BCF (body–caudal-fin) and MPF (median–paired-fin) modes, arguing for the stability advantages of undulatory thrust in complex environments. To further enhance energy efficiency and maneuverability, Zhang (2024) [55] surveyed the application of Computational Fluid Dynamics (CFD) in optimizing biomimetic structures, such as shark-skin riblets and manta ray pectoral fins. Conversely, Tian (2025) [64] proposed a high-speed flapping mechanism based on elastic snap-through instability to significantly boost the instantaneous thrust of soft robotic fish. Addressing long-endurance missions, Wang (2025) [65] explored hybrid propulsion combining biomimetic hulls with buoyancy-driven gliding, analyzing key technologies such as ultra-light structures and thermal adaptation; similarly, Petritoli and Leccese (2025) [66] provided a comprehensive overview of hybrid underwater gliders incorporating biomimetic propulsion. Additionally, Zhang (2025) [67] reviewed the structural design and actuation bottlenecks of water-surface-hopping robots.

Regarding perception, control, and swarm intelligence, Wang (2024) [68] systematically reviewed the hierarchy from low-level CPG gait generation to high-level closed-loop strategies, identifying machine learning-augmented hybrid control as a future direction. Liu (2023) [69] demonstrated the feasibility of embedding visual recognition algorithms for automated tasks and proposed standardized performance metrics (e.g., speed coefficients) to facilitate cross-study comparison. In terms of swarm coordination, Zhao (2022) [70] elucidated formation mechanisms that exploit the Kármán vortex street for energy saving, while Ramesh (2026) [71] proposed a theoretical framework applying natural shoaling mechanisms to robotic swarms, discussing the transition from individual to collective intelligence.

Despite these extensive reviews covering structures, fluid dynamics, and control, no systematic taxonomy specifically for biomimetic manta ray robots has been established to date. Existing studies often focus on single technical dimensions, lacking a systems engineering perspective on how these robots adapt to physical environmental gradients ranging from shallow waters to full-ocean depths. This paper aims to fill this gap by establishing “Operational Depth” as the core axis. We construct a unified theoretical framework encompassing the “Full-Ocean-Depth Manta Ray Atlas—Structural Evolution—Energy Utilization—Control”. By systematically analyzing the paradigm shift from mechanism verification in shallow waters to pressure adaptation and energy optimization in the deep sea, this review provides theoretical support for the design of next-generation deep-sea operational platforms.

### 1.5. Research Scope and Paper Structural Organization

Based on the aforementioned background, this paper adopts a systems engineering perspective to comprehensively delineate the evolutionary logic of biomimetic manta ray robots from shallow-water mechanism verification to full-ocean-depth engineering application. This paper does not merely list prototypes but attempts to construct a unified theoretical framework encompassing the “Full-Ocean-Depth Manta Ray Atlas—Structural Evolution—Energy Utilization—Control,” analyzing the mutual constraints and synergistic mechanisms of various subsystems under the dominance of depth.

The full text is divided into six sections. Excluding this introductory chapter, the remaining chapters are arranged as follows:

Section 2: Global Genealogy—From Shallow-water Testing to Full-ocean-depth Challenges. This chapter uses “operational depth” as the axis to categorize global biomimetic manta ray technology into four technical lineages: shallow-water experiments, medium-shallow-sea operations, deep-sea exploration, and full-ocean-depth extremes. It focuses on how design paradigms shift from pursuing fluid mechanism replication as depth increases.

Section 3: Structure and Actuation—The Mechanical Paradigm of Rigid-Flexible Integration. This chapter delves into the materials and construction of the biomimetic body. By comparing the advantages and disadvantages of rigid and purely soft structures, it argues that “rigid-flexible coupling” is the optimal solution for resolving the contradiction between drive efficiency and load-bearing capacity under high deep-sea pressure.

Section 4: Energy Systems and Long-endurance Strategies. This chapter focuses on the “metabolic system” of the robot. It examines the nonlinear constraints imposed by deep-sea low temperature and high pressure on energy systems and explores the synergistic effects of pressure compensation technology, glide-flap hybrid drive mechanisms, and environmental energy harvesting (thermal, vibrational) in extending the operational radius.

Section 5: Motion Control—From Analytical Models to Swarm Intelligence Evolution. This chapter analyzes the evolutionary trajectory of control systems. It describes the transition from traditional methods dependent on precise mathematical models toward bio-inspired CPG control and ultimately toward data-driven control and decentralized swarm intelligence based on deep reinforcement learning.

Section 6: Summary and Outlook. This chapter summarizes the paper and provides an outlook on future directions for deep-sea biomimetic robots, including cross-medium penetration, bio-mechanical hybrid intelligence, and in situ deep-sea energy replenishment stations.

## 2. Taxonomy of Biomimetic Manta Ray Submersibles Governed by Operational Depth

### 2.1. Introduction

**Figure 3:**
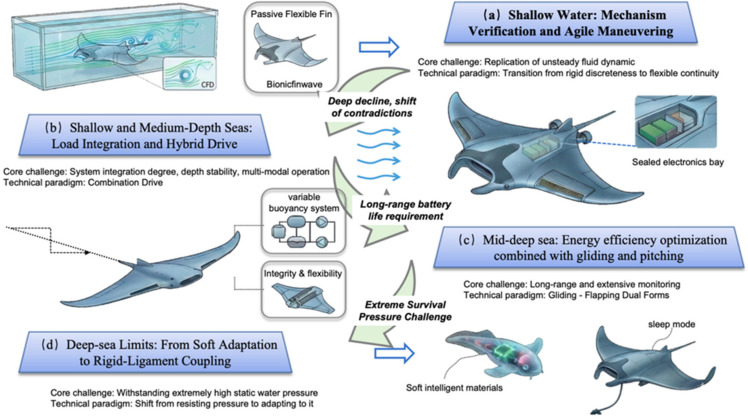
Technical lineages and evolutionary logic of biomimetic manta ray submersibles based on operational depth.

As shown in Figure 3, The developmental history of biomimetic manta ray robots is an engineering exploration journey governed by the core physical parameter of “operational depth”. Depth profoundly constrains the design of a robot’s structure, energy, and sensing systems by altering hydrostatic pressure, environmental fields, and propagation characteristics. As operational depth increases, the core design contradiction undergoes a fundamental shift: from the precise replication of biological unsteady hydrodynamics in shallow waters [72] toward the pursuit of survival under extreme pressure and long-endurance efficiency in deep-sea environments [73]. Consequently, global research achievements can be categorized into four technical lineages: shallow-water experiments, medium-shallow-sea operations, deep-sea exploration, and full-ocean-depth limits [74]. This chapter utilizes depth as the primary axis to systematically delineate the key bottlenecks and design paradigm shifts at each level. By comparing mainstream international technical routes, it analyzes the evolutionary logic of biomimetic propulsion technology as it transitions from the laboratory to the deep sea.

### 2.2. Shallow-Water Biomimetic Manta Ray Submersibles

As shown in Table 1, Shallow-water regions (typically <100 m in coastal zones or controlled tanks) are the core scenarios for validating the hydrodynamic mechanisms of biomimetic robots [53]. The primary challenge at this stage is to mechanically simulate the spatiotemporal deformation of biological pectoral fins to achieve peak propulsive efficiency in unsteady flow fields [25]. Regarding technical evolution, the field has undergone a paradigm shift from “rigid discrete” toward “flexible continuous” designs. Early rigid schemes utilized multi-link mechanisms to fit pectoral fin motion [75], Chu adopted a six-bar mechanism to simulate pectoral fin movement, validating the propulsive and lift performance of this rigid structure in shallow waters through experiments, which provided a kinematic benchmark for subsequent flexible refinements [76]. However, complex mechanical structures often result in additional parasitic mass and energy dissipation.

To overcome the limitations of rigid structures, recent research focus has shifted toward utilizing the passive deformation characteristics of soft materials. Wang proposed a soft manta ray robot driven by bidirectional dielectric elastomer (DE) artificial muscles [77,78], leveraging the rapid response of DE materials to significantly enhance swimming speed and maneuverability in complex shallow-water environments [79]. This “fluid-structure coupling” strategy utilizes the compliance of materials to induce vortex shedding, markedly reducing mechanical transmission costs. Addressing the gradient characteristics of biomimetic materials, Liu proposed a rigid-flexible coupled design based on multilayer soft materials to achieve efficient cruising [80]. Nevertheless, purely flexible designs also encounter environmental adaptability challenges. To address shallow-water wave disturbances, Liu combined soft materials to design a novel flapping-wing structure, focusing on analyzing and optimizing the robot’s motion response and structural stability even under wave conditions, thereby overcoming the weak current resistance of traditional soft fins [81]. Additionally, for miniaturization and stealth requirements, Chen utilized ionic polymer-metal composite (IPMC) artificial muscles to drive a micro-manta ray robot, achieving silent swimming at low voltages and providing early technical validation for covert shallow-water reconnaissance [82].

**Table 1:** Global taxonomy and performance characteristics of biomimetic manta ray robots based on operational depth.

	Shallow-Water Robots	Shallow-Water Test Platforms	Mid-to-Deep Sea Platforms	Full-Ocean Depth Platforms
**Operational Depth**	<100 m	100–300 m	300–1000 m	>1000 m
**Design Contradictions**	Reproduction of Unsteady Hydrodynamics	Payload Integration and Attitude Stability	Limited Energy vs. Long-Range Operation	Extreme Environment and Physical Limits
**Main Technical Routes**	① Rigid Discrete Control ② Flexible Passive Deformation	Hybrid Propulsion	Gliding-Flapping Dual Mode	① Soft Body Pressure Equalization ② Rigid Body Strategic Endurance
**Typical Cases**	① MantaDroid [72] ② BionicFinWave [83]	① BIOM ② Cyro [84]	① BOSS Manta Ray ② Manta Ray [74]	① Deep-Sea Soft Robot [73] ② Manta Ray UUV Program
**Technical Advantages**	① High Agility ② Kinematic Fidelity	① Engineering Practicality ②Capable of Carrying Modular Payloads	① Balances Long Range and Precise Operation ② Addresses High Energy Consumption of Continuous Flapping	① Soft Body: Mechanism Lightweighting ② Rigid Body: Enables Strategic Endurance
**Limitations**	① Poor Current Resistance ② Low Payload Capacity ③ Weak Environmental Perception	① High Noise ② Short Endurance	① Buoyancy Adjustment System Lag Prone to Depth Loss ② Bulky Structure	① High Communication Latency ② Severe Material Aging

At the optimization level of propulsion mechanisms, leveraging passive dynamic characteristics has become a key to enhancing efficiency [44]. The EGO unified design framework established by Liu achieved synergistic optimization of geometric and kinematic parameters [81]. Two distinct technical paradigms have emerged: a “minimalist” cruise mode pursuing extreme energy efficiency and an “omnidirectional maneuvering” mode for adapting to complex environments.

In the exploration of flexible passive deformation technical routes, the architecture of MantaDroid, developed by Chew at the National University of Singapore, completely abandoned complex servo motor arrays, utilizing only two high-torque motors to drive the leading edges of the pectoral fins; this employs the structure’s “mechanical intelligence” to share the computational load of the control system [85,86,87]. In contrast to MantaDroid, the BionicFinWave introduced by Festo represents another technical paradigm for operations in confined spaces [83], utilizing a lateral fin drive structure to achieve the reversibility of the “inverse Kármán vortex street,” endowing it with the rare biological capability of “backward swimming” [79,83].

As application scenarios expand, shallow-water biomimetic robots are transitioning from simple “mechanism replication” toward “intelligent operations” with environmental perception capabilities. The Northwestern Polytechnical University team achieved efficient drive based on CPG models; Cao used a CPG to control the phase and frequency of flexible pectoral fins, achieving realistic biological swimming postures through neural network adjustment and resolving the coordination control problem of multi-degree-of-freedom soft structures [88,89]. Integrating multimodal locomotion and sensing systems has become a current research hotspot; Zhang designed a dual-mode manta ray robot with “seabed walking” capabilities, achieving stable residency and fine operations in shallow-water benthic environments by merging pectoral fin flapping with abdominal leg motion [90]. More critically, addressing the challenge of underwater vision degradation, Zhao developed a vision positioning system based on an improved YOLOx algorithm for complex lighting in shallow waters, significantly enhancing the target detection accuracy and relative positioning capability of biomimetic robots in dynamic water environments, marking the gradual resolution of the “sense-compute-control” loop for shallow-water biomimetic robots [91].

### 2.3. Medium-Shallow Sea Biomimetic Manta Ray Submersibles

As operational depth extends to the 100–300 m range on the continental shelf edge and mesopelagic waters, the role of biomimetic robots undergoes a fundamental shift—transforming from “mechanism verification prototypes” in the laboratory into “transport platforms with practical operational capabilities”. The physical environment at this depth presents significant complexity: exponential attenuation of light renders visual navigation ineffective, the presence of thermoclines interferes with sonar propagation, and the hydrostatic pressure of 1–3 MPa requires reliable pressure-resistant sealing for core electronic bays [73]. Consequently, the primary design metrics at this stage shift from purely pursuing propulsive efficiency (CoT) toward system integration, depth-keeping stability, and multimodal operational capability.

To address the contradiction between payload integration and endurance, international technical schools have gradually converged toward “hybrid propulsion”. Although pure pectoral fin flapping possesses advantages in cruising energy efficiency, it faces power saturation issues in low-speed hovering, fine attitude adjustment, and resisting strong background currents (>1 m/s). To this end, researchers have proposed an engineering paradigm that combines traditional propellers or buoyancy engines with biomimetic flexible wings. This “dual-modal” design aims to compensate for the deficiencies of pure biomimetic forms in steady-state control through active control technology, marking the entry of biomimetic robot R&D into the systems engineering phase [92].

In the fields of military reconnaissance and high-precision mapping, platform stability is critical to the quality of sensor data. The Flimmer and its derivative platforms developed by the U.S. Naval Research Laboratory (NRL) epitomize the design philosophy of “fusing vector propulsion with biomimetic forms”. Kahn pointed out that the pulsed thrust generated purely by pectoral fin flapping causes periodic pitch and roll oscillations of the carrier; such high-frequency attitude jitter is fatal to the imaging quality of side-scan sonar and synthetic aperture sonar (SAS). To achieve steady-state cruising, the platform adopts a unique decoupled drive layout: the main body relies on a pair of high-aspect-ratio biomimetic pectoral fins for long-distance efficient cruising, while a tiltable vector water-jet thruster is integrated at the tail. Furthermore, addressing fixed-depth cruising requirements in medium-shallow seas, he introduced an S-plane control algorithm, effectively resolving the depth-keeping problem of biomimetic manta rays under variable buoyancy drive [93].

Conversely, addressing the demand for large payloads in long-endurance environmental monitoring, the Cyro robot developed by Virginia Tech extended the boundaries of biomimetic platforms from the dimension of “size effect”. As a large biomimetic autonomous underwater vehicle weighing over 70 kg, Cyro operates in a high-Reynolds-number flow field (Re > 10^5^), where fluid inertial forces play a dominant role [94]. Research by Villanueva showed that with an increase in characteristic scale, the fluid added-mass effect becomes more pronounced [84]. The team used linear actuators to drive massive silicone wing surfaces, confirming that large flexible bodies can more effectively utilize the reaction force generated by wake vortex shedding during low-frequency, large-amplitude flapping, achieving extremely high energy conversion efficiency. However, this stage still faces the contradiction between “energy density and acoustic stealth”. Cavitation noise generated by rotating machinery (propellers/pump-jets) in hybrid drives during high-frequency operation weakens the acoustic stealth advantage of biomimetic robots to some extent. Future improvements lie in developing silent artificial muscles based on electroactive polymers (EAP) or combining reinforcement learning to optimize the gliding trajectory of variable buoyancy systems (VBS) to achieve wide-area coverage of medium-shallow sea areas while maintaining silent characteristics [95].

### 2.4. Mid-Deep Sea Biomimetic Manta Ray Submersibles

When the operational depth reaches the 300–1000 m range in mid-deep seas, the mission profile of biomimetic robots changes fundamentally. This region is a critical battlefield for subsea pipeline inspection, mine countermeasures (MCM), and mesopelagic hydrological monitoring. Unlike shallow-water missions emphasizing instantaneous maneuverability, mid-deep-sea missions require endurance for several weeks and an operational radius covering hundreds of kilometers. In this context, the energy cost of relying purely on high-frequency flapping-wing propulsion increases sharply.

Consequently, the research focus at this stage has shifted from “precise replication of biological movement” toward “utilizing biological appearance to achieve extreme energy efficiency management”. This shift catalyzed the rise of “gliding-flapping dual-mode” technology, which utilizes the manta ray’s flat and wide streamlined body as a high-lift-to-drag-ratio lifting surface [96], combining the advantages of buoyancy drive (gliding mode) and biomimetic propulsion (flapping mode) [74].

**Figure 4:**
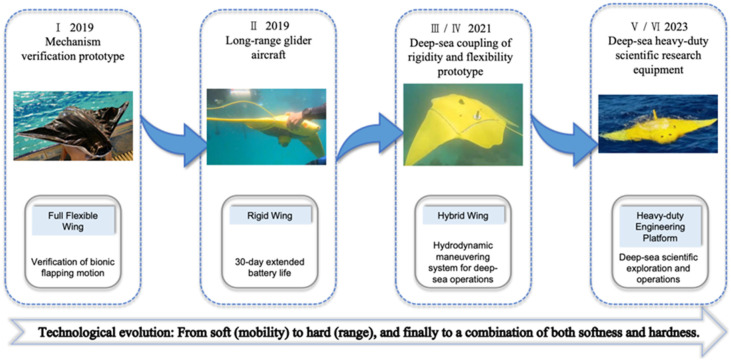
Evolutionary lineage of biomimetic manta ray submersibles from Northwestern Polytechnical University (NPU). The figure shows the iterative path of six generations of prototypes: (I) Mechanism Verification (2019): Developed a fully flexible wing prototype (Type I), validating the flexibility of low-frequency flapping propulsion; (II) Long-endurance Breakthrough (2019): Introduced a rigid gliding wing design (Type II), solving the energy challenge for 30-day/1000 km endurance; (III/IV) Rigid-Flexible Coupling (2021): Explored rigid-flexible integration for deep-sea environments, achieving 1000 m-class submergence (Type IV); (V/VI) Heavy-duty Engineering Application (2023): Finalized as a large-scale, heavy-payload deep-sea scientific research platform (Type VI), achieving the unification of full-ocean-depth capability and biological affinity.

As shown in Figure 4 and Table 2, As a pioneer in academic research in this field, the Northwestern Polytechnical University (NPU) team has constructed a complete genealogical technical system from shallow-water mechanism verification to deep-sea engineering applications through the iterative development of “six generations of prototypes” [28,70,97,98]. The Type I submersible developed in 2019, as a lightweight verification platform, weighed only 10 kg with a wingspan of 0.8 m. It adopted a fully flexible wing structure and achieved 5–8 h of endurance through 0.5 Hz low-frequency flapping, validating the flexibility of biomimetic propulsion in shallow waters [99]. The subsequent Type V (2022), while maintaining the fully flexible wing design, increased the weight to 30 kg and the upper limit of flapping frequency to 0.8 Hz, integrating vector hydrophones and Doppler velocity loggers to demonstrate its application potential in water quality monitoring and acoustic detection. To resolve the energy bottleneck of deep-sea long-distance operations, the team innovatively introduced gliding mechanisms into medium-sized platforms. The Type II (2019) adopted a fully rigid wing design with a wingspan of 2.0 m and a weight of 120 kg, validating the feasibility of achieving 30-day/1000 km long endurance through gliding at a depth of 500 m. Meanwhile, the concurrent Type III (2020) returned to a fully flexible wing design (100 kg, 2.0 m wingspan) under the same submergence and endurance metrics, further balancing gliding efficiency with biological affinity. Iteration of this technical route achieved a breakthrough in Type IV (2021); the weight of this model jumped to 500 kg with a wingspan of 3.0 m, adopting a semi-flexible wing structure. It successfully extended the operational depth to the 1000 m level and possessed deep-sea operational capabilities for carrying scientific research equipment such as CTDs [100].

Representing the highest level of this technical lineage is the Type VI biomimetic manta ray submersible launched in 2023. This model has a total length and wingspan reaching the 3 m and 4.2 m levels, respectively, with a weight in air of up to 800 kg and a payload capacity of 50 kg. Type VI broke through the challenge of fully flexible wing drive under large-scale structures, achieving efficient cruising at a speed of 2–3 km in 1000 m deep sea by simulating the flexible deformation of manta ray pectoral fins. In addition, Type VI integrates a high-performance control system and multi-source optical, acoustic, and magnetic sensors, capable of executing complex deep-sea exploration tasks.

In the field of heavy-tonnage defense, the DARPA “Manta Ray” project of Northrop Grumman represents the path of industrial-grade rigid platforms. The project aims to develop high-payload, ultra-long-endurance unmanned submersibles, primarily achieved through buoyancy-driven gliding and propeller propulsion. In February and March 2024, the “Manta Ray” prototype completed full-scale underwater testing off the coast of Southern California, validating its hydrodynamic performance and the effectiveness of multiple propulsion modes (buoyancy, propeller, control surfaces). Its design focus lies in utilizing the geometric advantage of the flat biomimetic shape when carrying large array sonars, as well as stability during seabed landing. Furthermore, to address long endurance, the project explored “seabed anchoring and hibernation” technology, allowing the submersible to reside long-term in a low-power state and achieve theoretical “infinite endurance” through innovative energy recovery systems such as ocean thermal energy.

### 2.5. Deep-Sea Biomimetic Submersibles

**Figure 5:**
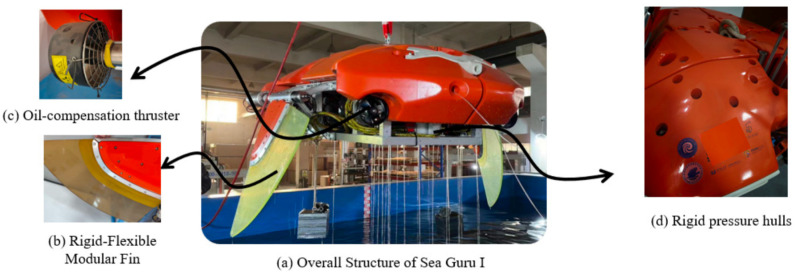
Electromechanical system composition and field performance validation of the Sea Guru I biomimetic submersible. (**a**) Overview of the submersible structure; (**b**–**d**) decomposition of key subsystems, including rigid-flexible modular pectoral fins, oil-compensated thrusters, rigid pressure hulls.

As shown in Figure 5, As operational depth breaks 1000 m and advances toward the 6000 m or even 11,000 m full-ocean depth, the physical environment undergoes a qualitative mutation. Hydrostatic pressure of 60–110 MPa is sufficient to crush conventional mechanical seal structures, causing traditional metal pressure hulls based on “resistive” designs to fall into a “weight-buoyancy” death spiral. Consequently, research in the full-ocean-depth stage presents the most disruptive technical revolution: the design philosophy shifts from “resisting pressure” toward “adapting to pressure” (pressure equalization), and the structural form shifts from “rigidly closed” toward “softly open” or “rigid-flexible heterostructured”.

**Figure 6:**
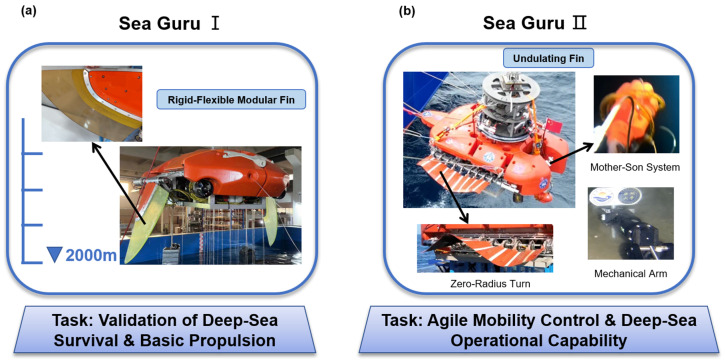
Iterative evolution of the “Sea Guru” series deep-sea biomimetic submersibles. (**a**) First-generation prototype Sea Guru I: Utilizes rigid-flexible modular pectoral fins, primarily for validating structural survival and basic propulsion at 2000 m depth; (**b**) Second-generation prototype Sea Guru II: Integrates undulating fins, a mother-son coordination system, and an underwater robotic arm to achieve zero-radius turns, agile maneuvering, and fine operations in complex deep-sea environments.

As shown in Figure 6, In this extreme environment, technical routes have primarily diverged into three frontier directions: first, the “fully soft intelligent materials” school, which advocates for utilizing the principle of pressure equalization to achieve extreme miniaturization and hadal survival of robots by eliminating rigid cavities [101]; second, the “rigid-flexible coupled structure” school, which advocates for simulating the skeletal features of cartilaginous fish and achieving the engineering implementation of deep-sea operations through the combination of local pressure resistance and flexible skin while maintaining fluid aerodynamic shapes; third, the “ultra-large strategic residency” school, which advocates for maintaining industrial-grade rigid architectures but resolving infinite endurance issues through the geometric advantages of biomimetic shapes and hibernation technology.

**Figure 7:**
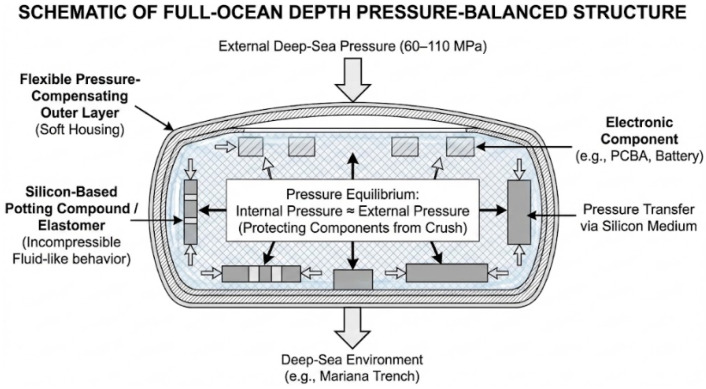
Schematic of full-Ocean Depth Pressure-Balanced Structure.

At one extreme end of hadal exploration, the collaborative results between Professor Li Tiefeng’s team at Zhejiang University and the Institute of Deep-sea Science and Engineering, Chinese Academy of Sciences, represent a breakthrough in materials science. Addressing the extreme pressure of 10,900 m in the Mariana Trench, the team abandoned traditional metal pressure hulls and instead drew inspiration from the non-enclosed skeletal structure of the deep-sea snailfish’s head [73]. Li proposed a “decentralized pressure-resistant electronic system” design method; As shown in Figure 7, the core logic lies in dismantling core components such as micro-control units (MCUs), batteries, and high-voltage amplifiers from centralized circuit boards, distributing them discretely and encapsulating them directly in a silicone matrix with excellent insulation and elasticity. This design achieves “pressure equalization,” allowing the internal medium pressure of the robot and the external deep-sea pressure to maintain a dynamic balance at all times, fundamentally avoiding structural implosion caused by pressure differences. At the drive level, the robot uses dielectric elastomer artificial muscles (DEA) to convert electrical energy into mechanical energy, overcoming the challenges of soft material hardening and dielectric breakdown under high pressure and low temperature.

As shown in Table 3, The “Sea Guru” series biomimetic manta ray submersibles developed by the team of Fan Dixia and Cui Weicheng from Westlake University demonstrated an engineering leap from “accessibility” toward “mobility” and “usability” in 2000 m-level deep-sea operations [102]. Sea Guru-I, which completed sea trials in the South China Sea in 2023, is the world’s first biomimetic structure-driven submersible to reach a depth of 2000 m. The submersible is 3 m long with a wingspan of 3.53 m and weighs approximately 741 kg in air. It adopts a modular design concept, integrating biomimetic hydrodynamic advantages and pressure-resistant engineering technology [103]. In terms of overall configuration, Sea Guru-I mimics the flat dorsal-ventral streamlined body characteristics of a manta ray to reduce fluid resistance and enhance stability. Its core propulsion mechanism adopts a set of rigid-flexible coupled flapping-wing systems driven by a deep-sea motor with an output torque of 87.5 Nm. Approximately two-thirds of the wingspan structure is a rigid leading edge used to provide necessary structural support and force transmission; the remaining part is a flexible trailing edge allowing for passive deformation. This rigid-flexible coupled design not only simulates the motion characteristics of real organisms but also effectively generates thrust by generating inverse Kármán vortex streets, significantly enhancing underwater propulsive efficiency. Furthermore, to compensate for the deficiencies of pure biomimetic propulsion in certain modes, the platform is auxiliary configured with two tail propellers for forward/backward swimming and steering, as well as three vertical propellers for vertical motion and pitch adjustment, achieving multimodal maneuverability control.

On the basis of Sea Guru-I successfully achieving deep-sea “accessibility,” the team of Fan Dixia and Cui Weicheng from Westlake University launched the second-generation biomimetic submersible Sea Guru-II, aiming to further resolve the “mobility” and “usability” challenges of deep-sea robots. Sea Guru-II is equipped with a novel undulating fin system. This design completely abandons the traditional rigid leading-edge structure, utilizing the phase difference control of multiple fin rays to simulate the wave-like oscillation of real fish. This not only further reduces hydrodynamic noise but also significantly enhances propulsive efficiency and maneuverability. Thanks to this propulsion system, Sea Guru-II possesses the capability for rapid stops, zero-radius turns, and maintaining attitude stability in complex flow fields, similar to real organisms, and is described as being able to operate as flexibly as a “fish in water” in the deep sea. In addition, the platform achieved breakthroughs in energy and payload, successfully applying a small Stirling engine for in situ power generation in an oxygen-free 1100 m deep-sea environment for the first time, and integrated a “mother-son” deployment system capable of releasing micro-unmanned aerial vehicles for narrow-space exploration.

### 2.6. Chapter Summary

This chapter reveals the fundamental laws of technical evolution driven by the core physical parameter of “operational depth” by sorting through the global technical lineage of biomimetic manta ray robots. As exploration depth descends, the design focus exhibits a clear paradigm shift across four levels.

At the shallow-water experimental stage (<100 m), research focuses on the “replication of unsteady hydrodynamic mechanisms”. Through the two routes of rigid discrete and flexible continuous designs, the mechanical simulation of complex flapping-wing motion and thrust generation issues were resolved, establishing the theoretical cornerstone for agile maneuverability. As operational depth descends to the medium-shallow sea platform stage (100–300 m), the core contradiction shifts toward “payload integration and steady-state control”. The introduction of hybrid propulsion strategies effectively compensates for the deficiencies of pure biomimetic propulsion in current resistance and hovering, achieving a transformation into an engineered transport platform. Entering the mid-deep sea operational stage (300–1000 m), faced with long-endurance requirements, research focus shifts toward “extreme energy efficiency management” [54]. The gliding-flapping dual-mode (e.g., the NPU lineage) has become the mainstream technical route to balance long-distance cruising and fixed-point fine operations. Finally, at the hadal extreme stage (>1000 m), faced with extreme hydrostatic pressure, technical paradigms undergo disruptive differentiation. These include the “fully soft pressure adaptation” school represented by Zhejiang University, the “strategic residency and hibernation” school represented by DARPA Manta Ray, and the “rigid-flexible coupling” scheme of Westlake University, which provides an engineering bridge between the two.

In summary, technical development is moving from individual rigid or soft bodies toward integration. Future hadal biomimetic robots will trend toward “rigid-flexible integrated” forms—utilizing soft materials to protect sensitive components for pressure resistance, relying on rigid skeletons to maintain fluid shapes and transmit thrust, and combining environmental energy harvesting technology to ultimately achieve the unification of full-ocean-depth, long-endurance, and highly intelligent operations.

## 3. Structure of Biomimetic Manta Ray Submersibles

### 3.1. Introduction

The structural design of a biomimetic manta ray robot is not merely the construction of a mechanical shell but a physical reconfiguration of the complex “fluid-structure-actuation” coupling system. As the physical interface connecting the internal energy system with the external deep-sea flow field, the structural characteristics of the robot directly dictate the efficiency of energy transfer and the response patterns of its fluid dynamics [104].

**Figure 8:**
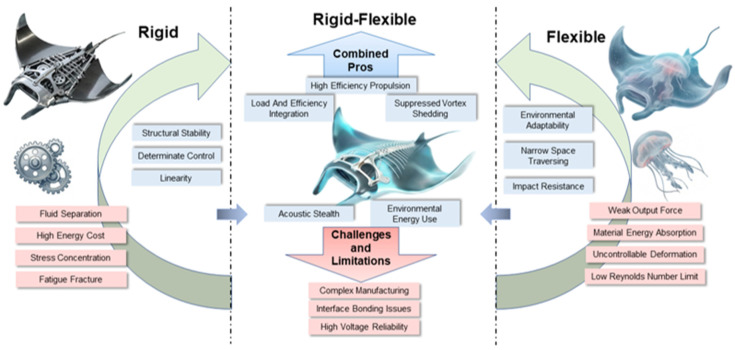
Technical characteristics of rigid, flexible, and rigid-flexible coupled structures.

As shown in Figure 8, In extreme deep-sea environments, structural design faces stringent constraints from multiple physical fields: on one hand, hydrostatic pressures on the order of 107 Pa require the structure to possess high stability to prevent buckling failure [73]; on the other hand, the cross-domain flow field characteristics, ranging from low to high Reynolds numbers, demand that the propulsion surface exhibits compliance similar to biological tissues to exploit unsteady hydrodynamic effects for high lift generation and flow separation suppression [105]. Traditional marine engineering design typically decouples “structural pressure resistance” from “propulsion”—employing high-strength titanium alloy hulls to resist pressure and rigid screw propellers to generate thrust. However, biological evolution demonstrates that deep-sea organisms, such as the manta ray, achieve an integration of load-bearing and efficient propulsion through the sophisticated fusion of skeletons (rigid support), muscles (active actuation), and skin (flexible membrane) [106].

This chapter focuses on the body structure of biomimetic manta ray robots, providing an in-depth analysis of the mechanical paradoxes encountered during the evolution from traditional rigid structures to soft structures. By comparing typical cases of different technical routes worldwide, we explore how “rigid-flexible coupling” strategies resolve the contradiction between payload and efficiency in engineering, and further analyze how frontier technologies, such as dielectric elastomers and self-healing materials [107], are reshaping the physical morphology of next-generation deep-sea robots.

### 3.2. Inefficiency of Rigidity and Deficiency of Flexibility

#### 3.2.1. Hydrodynamic Failure and Energy Traps of Biomimetic Rigid Structures

For a long time, the mainstream design of underwater vehicles has followed “rigid body dynamics” standards. Traditional torpedo-shaped AUVs or early biomimetic prototypes typically utilized metals or hard composites for their main bodies and propulsion surfaces. While this design ensures structural determinacy and control linearity, it proves extremely inefficient when handling complex deep-sea fluid interactions.

The primary issue is flow separation on rigid wing surfaces. Biomechanical research indicates that fish utilize fluid energy through the undulatory bending of their bodies; however, early man-made rigid pectoral fins were incapable of bending. From a fluid mechanics perspective, a completely rigid flapping wing or pectoral fin cannot produce effective spanwise or chordwise deformation in water. According to thin-airfoil theory, when a rigid wing flaps at a high angle of attack, the LEV becomes highly unstable and sheds prematurely, leading to a sharp drop in the lift coefficient and a surge in the drag coefficient. This not only reduces propulsive efficiency but also generates violent oscillatory moments, increasing the difficulty of attitude control. Experimental data directly support this: Heathcote and Gursul [108] found in water tunnel experiments on various flexible airfoils that moderate chordwise flexibility significantly improves LEV attachment time. Their research showed that within a specific stiffness range, the thrust coefficient (C_T) generated by a flexible wing was more than 40% higher than that of a rigid wing, with the peak propulsive efficiency shifting toward higher frequencies. More recent experimental data further confirm that under specific bending stiffnesses and pitching frequencies, airfoils equipped with flexible panels generate 2–4 times the thrust of purely rigid airfoils, and flexible leading edges effectively delay vortex shedding [109]. This suggests that rigid structures waste most of the propulsive gains potentially available from fluid interaction. Research by Marais [110] also noted that the average thrust of flexible hydrofoils is three times that of rigid ones, and flexibility significantly suppresses asymmetric wake breaking, thereby enhancing swimming stability.

A second issue involves high energy costs and low fluid utilization. Triantafyllou at MIT pointed out in a classic fluid mechanics review that the key to efficient biological swimming (e.g., tuna, manta rays) lies in controlling the oscillation frequency within a specific Strouhal number range. This allows the utilization of body flexibility to “capture” energy from the wake vortices rather than generating useless rotational kinetic energy like rigid propellers [104]. Winter, in developing the RoboClam, also revealed the limitations of rigid structures in complex environmental interactions. Although RoboClam was designed to simulate the burrowing behavior of razor clams, early rigid tests showed that when facing viscoelastic seabed sediments, the rigid structure could not reduce insertion resistance through deformation. Consequently, its energy consumption (CoT) increased exponentially with depth [111]. This phenomenon persists in biomimetic swimming: rigid pectoral fins attempt to “forcibly” push fluid aside rather than “complying” with it. This antagonistic interaction results in substantial energy loss through turbulent dissipation rather than conversion into effective thrust [112,113]. Moreover, rigid structures lack the damping characteristics of biological tissues; under structural micro-deformations caused by deep-sea high pressure, stress concentration at joints becomes significant, making them highly susceptible to fatigue fracture under long-term cyclic loading.

#### 3.2.2. Force Output Scarcity and Control Nonlinearity of Biomimetic Flexible Systems

To address the issues of rigid structures, scientists began researching soft robots [114]. These robots, typically composed of silicone, hydrogels, or elastic fabrics, attempt to achieve extreme environmental adaptability by mimicking the low-modulus characteristics of biological tissues [115]. While highly adaptable, purely soft structures have exposed weaknesses in power output during deep-sea propulsion tasks.

The primary challenge is energy absorption and thrust loss within soft materials. The core problem is that the material is too soft to transmit force effectively; the Octobot developed by Harvard’s Wyss Institute is a typical representative of this route [116]. As the world’s first fully soft, autonomously chemically driven robot, it demonstrates amazing narrow-space traversal and impact resistance, yet its propulsive force is extremely weak. Rus and Tolley noted in a review that without a rigid skeleton as a medium for force transmission, soft materials undergo isotropic volume expansion or deformation when actuators are triggered, causing the driving energy to be largely absorbed as internal energy by the material itself, making it difficult to output high-density directional thrust to the fluid [117].

Furthermore, purely flexible structures face severe risks of losing control due to passive deformation in fluids. Under the impact of high-speed ocean currents (>1 m/s), soft pectoral fins lacking rigid support undergo uncontrollable passive large deformations, leading to a failure of the angle of attack or even the generation of negative lift [118]. For deep-sea operations, this means the robot cannot maintain its position in currents, let alone execute scientific missions requiring precise positioning. The consensus in the international fluid mechanics community has gradually shifted: while soft materials represent the future, purely soft structures lacking skeletal support struggle to meet deep-sea cruising requirements at high Reynolds numbers [119].

### 3.3. Rigid-Flexible Coupled Structures of Biomimetic Manta Ray Submersibles

Faced with the binary dilemma of rigidity and flexibility, biological anatomy provides a perfect solution: the manta ray’s pectoral fin is not a uniform material but a gradient structure composed of a high-strength proximal cartilaginous skeleton (generating driving torque) and distal collagen-rich flexible connective tissue (optimizing fluid rectification). This “internally rigid, externally flexible; proximally hard, distally soft” construction has inspired the “rigid-flexible coupling” design approach in contemporary biomimetic robots [120].

#### 3.3.1. Variable Stiffness Mechanisms and Passive Regulation of Chordwise Flexibility

**Figure 9:**
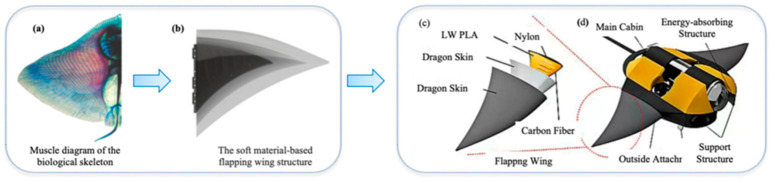
Design evolution of biomimetic soft submersibles from biological mechanisms to engineering implementation. (**a**) Skeletal and muscular anatomy of a biological manta ray pectoral fin, serving as the biological prototype; (**b**) model of a biomimetic flapping wing based on soft materials; (**c**) exploded view of the multilayered flapping wing composition, showing the composite distribution of lightweight PLA, nylon, Dragon Skin silicone, and carbon fiber; (**d**) overall system assembly of the robot, including the main cabin, external energy-absorbing structures, and support framework. Adapted from Ref. [80], used under CC BY 4.0.

As shown in Figure 9, The core of rigid-flexible coupling lies in the scientific distribution of stiffness to maximize propulsive efficiency. The current mainstream international technical route uses a rigid leading edge to establish the motion frequency and a flexible trailing edge to generate phase lag, thereby forming a perfect traveling wave propulsion.

The Hybrid Manta Ray Robot developed by Beihang University and international collaborators represents pioneering work in this field. The robot’s pectoral fins utilize a split design where the leading edge is driven by rigid carbon fiber rods that directly bear the motor’s torque output, while the trailing edge consists of high-performance membranes and embedded elastic skeletons. Experiments and CFD simulations showed that when the rigid leading edge flaps at a specific frequency, the flexible trailing edge produces a phase lag relative to the leading edge due to fluid inertial forces and elastic restoration forces. This passive deformation allows the pectoral fin to maintain an effective angle of attack during the downstroke, directing more of the fluid’s reaction force backward (thrust component) rather than downward (lift component) [121]. The research by Liu Y, Xie Y, Cui Z and others indicates that this design of the flexible and rigid coupled pectoral fin enables the robot to achieve a higher swimming speed and better stability at low-frequency flapping than a purely rigid fin [69,122,123]. Numerical simulations by Heathcote and Gursul further confirmed that chordwise flexibility has a significant impact on aerodynamic/hydrodynamic performance [110], showing that the propulsive efficiency of flexible flapping wings is much greater than that of rigid ones [108]. Research by Shyy also found that while increased flexibility may lead to a decrease in lift at low angles of attack, at high frequencies, flexibility can help thrust recover to near-rigid levels, demonstrating excellent broadband adaptability [124].

Propulsive efficiency is commonly measured by the Strouhal number (St). Dewey discovered through experiments that compared to rigid plates, flexible plates can achieve a 100–200% thrust amplification and an approximately 100% increase in propulsive efficiency, with the optimal efficiency occurring in the St range of 0.25–0.35 [24]. This range aligns perfectly with the optimal range for biological swimming. The soft robotic fish developed by Katzschmann successfully maintained St values between 0.25 and 0.35, achieving efficient cruising [44]. Thiria and Godoy-Diana pointed out that passive deformation allows flexible wings to maintain high propulsive efficiency over a broad frequency range, whereas rigid wings are effective only within a narrow band [125]. Furthermore, research by Lucas showed that by customizing the bending patterns of non-uniform flexible hydrofoils, flexible wings consume significantly less energy and achieve higher propulsive efficiency than rigid ones [126]. He experimentally investigated the spanwise flexibility of biomimetic pectoral fins, showing that the combination of high stiffness at the fin base and low stiffness at the tip significantly reduces lift and pitching moments while maintaining high thrust, which validates the decisive role of the stiffness gradient distribution of biological structures on propulsive performance [127].

#### 3.3.2. Gradient Material Distribution and Biomimetic Anisotropic Design

Beyond macroscopic structural assembly, technologies for rigid-to-flexible transition at the material microstructure level have also made breakthroughs in recent years. This technical route aims to eliminate the obvious physical interface between rigid skeletons and flexible skins, thereby resolving the issue of interface delamination caused by modulus mismatch under deep-sea high pressure.

Bartlett demonstrated the design concept of gradient structures based on multi-material 3D printing, achieving a continuous exponential gradient in stiffness by precisely controlling the material mixing ratio at the voxel level. Bartlett noted that this gradient material distribution eliminates stress concentration and makes the structure more stable under load [128]. This principle is equally applicable to the fin design of deep-sea robots. Research by Heathcote showed that moderate spanwise flexibility is crucial for flapping propulsion; experimental data indicated that the maximum thrust coefficient of flexible wings was more than 50% higher than that of rigid wings, while reducing input power [105]. Kang also found that spanwise flexibility not only increases thrust but also optimizes the effective angle of attack distribution through passive torsion [129].

In the field of glider design, the advantages of rigid-flexible integration are equally significant. Qin performed numerical simulations and noise characteristic research on a blended-wing-body (BWB) underwater glider, showing that the optimized BWB glider achieved a maximum lift-to-drag ratio (L/D) of 15.38, significantly higher than that of traditional rigid cylindrical gliders [130]. Furthermore, Hu showed that flexible membrane wings outperform rigid ones in average L/D during flapping flight [131]. Yi ‘s research further indicates that an uneven distribution of chordwise flexibility generates a higher thrust efficiency than uniform flexibility [132]. Shyy quantified the impact of such deformation, noting that when the chordwise flexible deformation amplitude is 0–0.5 times the chord length, it can generate wake structures beneficial for thrust [124].

### 3.4. Chapter Summary

This chapter provides an in-depth exploration of the design criteria for the body structure of biomimetic manta ray submersibles, emphasizing that structural design encompasses more than the construction of a mechanical shell; it represents a physical reconfiguration of the “fluid-structure-actuation” (FSA) coupling relationship. To address the mechanical paradox between structural pressure resistance and propulsive compliance in extreme deep-sea environments, this chapter first analyzes the energy traps encountered by traditional rigid structures due to the instability of the LEV during unsteady fluid interaction. Furthermore, it examines the risks of losing control in purely soft structures under high-Reynolds-number conditions, primarily caused by insufficient force output and control nonlinearity.

Drawing inspiration from biological anatomy, this chapter delineates the evolutionary path toward “rigid-flexible coupling”. By replicating the gradient characteristics of biological manta rays—specifically the “internally rigid, externally flexible; proximally hard, distally soft” construction—the design utilizes a rigid skeleton to establish the primary motion frequency while leveraging a flexible trailing edge to generate passive phase lag. This configuration allows the submersible to maintain the Strouhal number (St) within the biologically optimal range of 0.25–0.35, thereby achieving an increase in propulsive efficiency of over 100%.

Additionally, this chapter explores the engineering applications of functionally graded materials (FGM) and multi-material 3D printing technologies in eliminating stress concentration and preventing interface delamination under deep-sea high pressure. This review concludes that the profound integration of microscopic material gradients and macroscopic rigid-flexible coupling not only resolves the paradox between power output and environmental compliance but also establishes a robust physical foundation for the high-efficiency and long-life operation of biomimetic manta ray submersibles in complex deep-sea flow fields.

## 4. Energy Systems and Long-Endurance Strategies

### 4.1. Engineering Constraints of Deep-Sea Energy Systems

**Figure 10:**
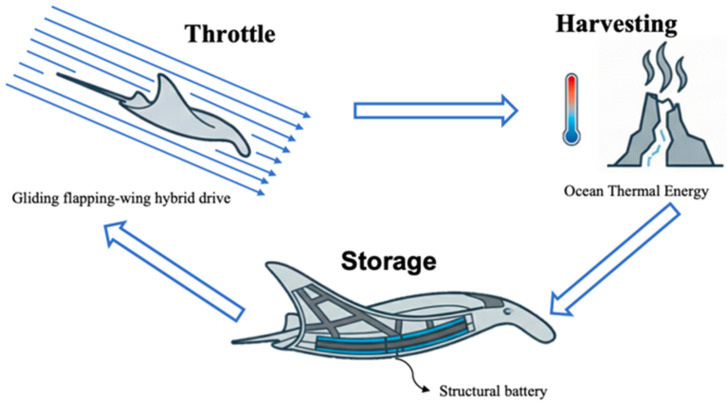
“Energy-saving, Storage, and Harvesting” strategies for deep-sea biomimetic manta ray submersibles.

As shown in Figure 10, In the system design of unmanned underwater vehicles (UUVs), energy capacity and endurance constitute the physical boundaries of the mission radius. Unlike terrestrial or shallow-water robots, the deep-sea environment (depth > 1000 m) imposes non-linear physical constraints on energy systems, leading to diminishing marginal utility for conventional designs. This chapter begins by examining the two core thermodynamic and mechanical bottlenecks facing deep-sea long-endurance missions: the structural weight penalty induced by hydrostatic pressure and the inhibition of electrochemical activity caused by low-temperature environments.

Deep-sea hydrostatic pressure increases linearly with depth. In traditional engineering paradigms, battery packs are typically encapsulated within pressure hulls made of titanium or high-strength aluminum alloys. According to thin-walled cylinder buckling theory, the wall thickness of the pressure hull must increase with the design depth to prevent instability. As noted by Stokey during the development of the REMUS 600, weight management of pressure-resistant structures is a central challenge in submersible design [133]. For full-ocean-depth submersibles such as the Nereus, expensive ceramic pressure hulls must be employed to mitigate structural weight [134]. This severe “dead weight” effect compresses the effective payload space for batteries, resulting in system-level energy densities for rigid pressure hull-based AUVs that are significantly lower than those of individual battery cells. Simultaneously, bottom-water temperatures in the deep sea remain consistently between 1–4 °C, which significantly suppresses chemical reaction rates. For mainstream lithium-ion batteries, low temperatures increase electrolyte viscosity and reduce lithium-ion diffusion coefficients, leading to a sharp rise in internal resistance and a decay in discharge capacity. Therefore, energy system design for biomimetic manta ray robots must extend beyond simple battery selection; it requires a system-level coupling optimization strategy that encompasses hydrodynamic drag reduction, pressure-resistant structural integration, and in situ energy replenishment [135,136,137,138].

### 4.2. Energy Saving on the Propulsion Side

Given that battery energy density is unlikely to break through physical limits in the short term, reducing the Cost of Transport (CoT) remains the most effective means of extending endurance. The unique flat, streamlined body and flexible pectoral fins of the manta ray provide a natural geometric foundation for hydrodynamic optimization. While traditional propeller-driven AUVs generally exhibit high CoT, underwater gliders (e.g., Slocum Glider) utilize environmental thermal energy or buoyancy regulation to achieve exceptionally low energy consumption [139]. To combine the high efficiency of gliders with the superior maneuverability of biological swimming, Zhang proposed a novel biomimetic manta ray robot design that integrates gliding and flapping propulsion [74]. This design achieves seamless switching between two modes by integrating a VBS and a center-of-gravity adjustment mechanism. During long-distance cruising, the robot locks its flexible pectoral fins, adjusts the VBS oil bladder volume to change net buoyancy, and utilizes its body as a lifting surface to glide along a sawtooth trajectory. When approaching a target area or requiring obstacle avoidance, the system switches to flapping mode to obtain high instantaneous thrust and maneuverability.

However, this hybrid propulsion strategy still faces control challenges in practical applications. Zhang (2022) pointed out that while the gliding mode is energy-efficient, its maneuverability is poor, making it difficult to cope with sudden complex current disturbances. Furthermore, the hydrodynamic transients during mode switching can cause attitude instability, requiring sophisticated non-linear control algorithms for a smooth transition, which increases the computational energy burden on the onboard processing units [74].

In the flapping propulsion mode, unsteady hydrodynamic efficiency depends on the matching between kinematic parameters and the fluid environment. Triantafyllou, in their foundational work, established the Strouhal number (St) as the core dimensionless parameter for measuring swimming efficiency, noting that propulsive efficiency peaks when St falls between 0.25 and 0.35 [140]. Subsequent studies further revealed the critical role of flexible structures; Fish and Lauder reviewed mechanisms through which organisms utilize passive and active fluid control to optimize propulsion [106]. Through systematic scaling law research, Dewey found that thrust generation is closely related to the stiffness of the flexible plate; when the driving frequency approaches the resonance frequency of the fluid-structure coupling system, maximum thrust can be generated with minimum energy consumption [24]. Engineering implementations by Zhang demonstrated that driving flexible pectoral fins via CPG control algorithms can effectively simulate these biological deformation characteristics, thereby optimizing the hydrodynamic response [141].

Despite the relative maturity of theoretical models, precisely maintaining the optimal St in engineering remains highly challenging. Research by Dewey showed that the high-efficiency propulsion window for flexible wings is narrow; if the material’s natural frequency does not match the environmental flow field, propulsive efficiency drops sharply [24]. Additionally, Triantafyllou noted that real-time sensing of flow velocity and adjustment of flapping frequency in turbulent sea conditions impose extreme requirements on sensor precision and response speed [140].

### 4.3. Energy Saving on the Storage Side: Structural-Functional Integration

To overcome the bottleneck of structural weight, modern deep-sea energy systems are undergoing a paradigm shift from “resisting pressure” toward “adapting to pressure” (pressure equalization), characterized by “pressure-hull-less” designs. Pressure-balanced oil-filled (PBOF) technology is currently the most mature engineering solution for kilometer-class submersibles. Song conducted detailed research on compensator design and oil characteristics in PBOF technology, pointing out that this method can effectively balance internal and external pressure differences, thereby eliminating the need for heavy pressure hulls [142]. As early as 2006, Wilson and Bales developed practical pressure-tolerant lithium battery packs, validating the feasibility of placing pouch cells in insulating oil to directly withstand deep-sea pressure [143]. For biomimetic manta ray robots, the advantage of PBOF technology lies in its geometric adaptability; pouch cells and oil bladders can be manufactured into flat, irregular shapes and dispersedly embedded within the internal space of the manta ray’s wide pectoral fins, significantly enhancing internal space utilization.

However, PBOF systems also have distinct limitations. Song noted that oil-filled systems add maintenance complexity, and the mass of the insulating oil itself reduces the overall specific energy density of the battery pack [142]. Furthermore, during long-term deep-sea operations, flexible compensation membranes face risks of fatigue and aging; any oil leakage not only leads to equipment failure but also results in marine environmental pollution [142].

Representing the frontier of next-generation deep-sea energy technology, all-solid-state batteries (ASSBs) and structural battery technologies exhibit immense application potential. A review by Zhang presented a counterintuitive conclusion: the deep-sea high-pressure environment actually benefits the performance of all-solid-state batteries because external pressure improves the interfacial contact between the solid electrolyte and the electrodes [144]. This characteristic makes ASSBs an ideal choice for deep-sea environments. Furthermore, Asp and Greenhalgh defined the concept of structural power composites, where materials simultaneously fulfill mechanical load-bearing and electrical energy storage functions [145]. Thomas further developed multifunctional structural-battery composites for marine systems [146]. Based on these cutting-edge technologies, future biomimetic manta rays could laminate solid-state battery layers into carbon fiber composite skins, achieving a “fuselage-is-battery” self-sustaining design that theoretically reduces parasitic mass to zero.

Nevertheless, this technology remains in the laboratory stage. Zhang emphasized that while high pressure improves interfacial contact, ASSBs still face technical bottlenecks in large-scale fabrication processes and cycle life [144]. Simultaneously, Thomas mentioned that the electrochemical safety of structural batteries after sustaining impact loads requires further validation, and repair costs after damage are far higher than those for traditional modular batteries [146].

### 4.4. Energy Replenishment: From Carried Energy to In Situ Energy Harvesting

Beyond increasing the energy carried for a single mission, constructing underwater energy replenishment networks and environmental energy harvesting systems is the ultimate solution for “infinite endurance”. To extend operational cycles, underwater wireless power transfer (WPT) technology has become a research hotspot. Regarding system integration, Feezor validated autonomous homing technology based on electromagnetic guidance [147], while Kojiya demonstrated the feasibility of non-contact automatic charging stations [148]. Leveraging the flat abdominal features of the manta ray, large-area receiving coils can be designed, allowing the robot to adhere to seafloor base stations like benthic organisms for high-efficiency inductive charging.

However, the underwater environment poses severe challenges to WPT efficiency. A review by Yu pointed out that the conductivity of high-salinity seawater causes significant eddy current losses, markedly reducing energy transfer efficiency [149]. Mohsan further added that inductive charging is extremely sensitive to coil alignment; under the interference of deep-sea currents, achieving millimeter-level autonomous docking and positioning is technically difficult [150].

Utilizing ocean thermal and mechanical energy for in situ replenishment is also a critical developmental direction. Webb successfully applied thermal engines in SLOCUM gliders, utilizing seawater temperature differences to drive buoyancy regulation [139]. Wang further reviewed the application of OTEC in UUVs, noting that phase-change materials can provide both propulsion and electricity for electronic equipment [151]. Additionally, for micro-power sensors, Han developed a triboelectric nanogenerator (TENG) based on fish gelatin, capable of harvesting mechanical energy from water flow fluctuations or biological movement [152].

While environmental harvesting technologies have broad prospects, their power density is generally low. Data from Han showed that the current output power of TENG can only drive sensors or low-power communication modules and cannot provide continuous energy for propulsion systems [152]. Wang also pointed out that the efficiency of OTEC systems is limited by the vertical temperature gradient of the sea area, possessing practical value only in specific tropical waters, which significantly restricts the operational range of the robot [151].

### 4.5. Chapter Summary

This chapter systematically discusses the pathways for achieving long-endurance capabilities in deep-sea biomimetic manta ray robots, constructing a multi-dimensional coupling optimization framework encompassing energy consumption, storage, and replenishment. Addressing the physical limits imposed on energy systems by deep-sea high pressure and low temperature, this chapter first clarifies the mechanism of “dead weight effects” and energy attenuation caused by conventional pressure-resistant encapsulation. It then proposes energy-saving strategies based on biomimetic hydrodynamics, effectively reducing the Cost of Transport (CoT) through the optimization of gliding-flapping hybrid propulsion modes and the Strouhal number (St).

Regarding storage architecture, this chapter explores the engineering paradigm shift from PBOF technology toward all-solid-state structural batteries, emphasizing forward-looking “structure-energy integration” designs to eliminate redundant mass and achieve a “fuselage-is-battery” configuration. Finally, this chapter analyzes the in situ replenishment mechanisms of underwater wireless power transfer and ocean environmental energy harvesting (thermal and mechanical). The review indicates that only through the deep decoupling and synergistic optimization of fluid, structural, and energy systems can the physical boundaries of deep-sea mission radii be fundamentally transcended, providing the power guarantee for the long-term autonomous evolution of biomimetic manta ray robots.

## 5. Motion Control

### 5.1. Introduction

**Figure 11:**
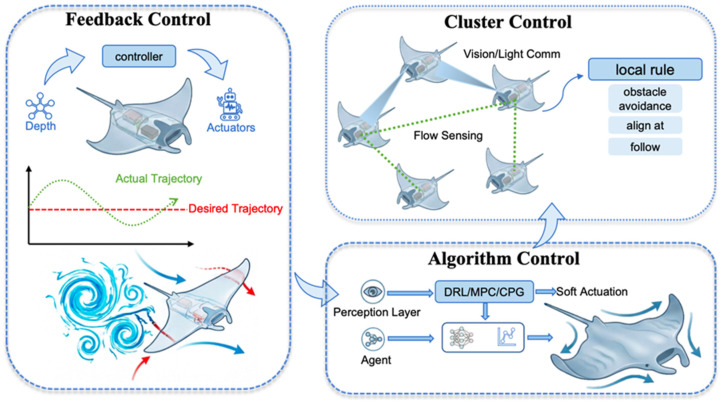
Control evolution framework for biomimetic manta ray submersibles from single-machine feedback to swarm intelligence.

As shown in Figure 11, As a typical high-aspect-ratio flapping-wing submersible, the motion control of the biomimetic manta ray represents one of the most challenging problems in the field of underwater robotics. A review by Hasan pointed out that, unlike traditional autonomous underwater vehicles (AUVs) based on propeller propulsion, the biomimetic manta ray does not possess a decoupled thruster configuration; instead, it generates unsteady lift and thrust through the complex deformation of flexible pectoral fins and their interaction with the fluid [153]. While this unique propulsion mechanism endows the robot with high maneuverability and biological stealth, it also poses severe challenges for control system design [154].

First, the system dynamics are characterized by high nonlinearity and strong coupling. The motion of the manta ray robot is inherently a coupled system of rigid-body dynamics and fluid dynamics [15]. The flapping of the pectoral fins not only produces propulsive force but also induces periodic fluctuations in pitch and yaw moments. Furthermore, as the robot’s forward speed, flapping frequency, and angle of attack change, the fluid added mass and damping coefficients exhibit significant time-varying characteristics, which are difficult to describe accurately using traditional linear hydrodynamic coefficients.

Second, actuator constraints and underactuated characteristics limit the controllable domain. Deep-sea high-pressure environments require actuators to possess high power density. Xu pointed out that although smart materials such as SMA more closely resemble biological muscle characteristics, they still exhibit nonlinear hysteresis in force output and response speed [155], making control based on precise models exceptionally difficult [156]. Meanwhile, biomimetic manta rays are typically underactuated systems; the dimension of control inputs (usually the frequency, amplitude, and phase of the pectoral fins) is much smaller than the dimension of the robot’s state space (position and attitude). This means that independent control of all DOFs is impossible, and specific maneuvers must rely on dynamic coupling.

Finally, the uncertainty of the unstructured deep-sea environment presents a significant hurdle. In the absence of external global positioning (e.g., GPS) and under conditions of high latency and low bandwidth in acoustic communication, the robot must rely on proprioception to cope with external disturbances such as internal waves and currents. Therefore, the control architecture must possess extreme robustness and adaptability. This chapter departs from traditional classification methods and, following the logic of engineering implementation, explores motion primitive generation (kinematic layer), dynamics and disturbance rejection control (dynamic layer), and learning-based strategy optimization (intelligent layer), with a focus on resolving the challenges of long-endurance gliding-flapping hybrid propulsion [21] and Sim-to-Real policy transfer.

### 5.2. Motion Primitive Generation: CPG and Kinematic Dimension Reduction

The primary task of biomimetic control is to simplify high-dimensional continuum deformation into controllable low-dimensional inputs. Biological research indicates that manta ray swimming relies on rhythmic signals generated by central pattern generators (CPGs) in the spinal cord. In his foundational review, Ijspeert established the core position of CPGs in biomimetic robot control, noting that utilizing mathematical oscillator models (such as Hopf or Kuramoto) can effectively resolve the coordination control problem of multi-DOF systems [157], driving pectoral fins to produce coupled spanwise and chordwise undulations [158].

#### 5.2.1. CPG Topology Design for High-Aspect-Ratio Pectoral Fins

Unlike elongated fish (e.g., eels, tuna) that primarily rely on body-wave propulsion, the manta ray utilizes massive pectoral fins for MPF mode swimming. In engineering implementation, this is typically modeled as a network of coupled oscillators distributed along the span of the pectoral fins.

Mainstream CPG models [159] mostly adopt Hopf or Kuramoto oscillators as core units. For biomimetic pectoral fins with multi-stage skeletons, the control model is usually constructed as a bilaterally symmetrical chain topology. The desired angle θ_i_ for each joint is determined by the oscillator state equations:

(1) $${\dot{r}}_{i}=\alpha \left(R_{i}-r_{i}\right)$$

(2) $${\dot{\phi }}_{i}=2\pi f_{i}+\sum w_{ij}\sin(\phi_{j}-\phi_{i}-\Delta \phi_{ij})$$

(3) $$\theta_{i}=r_{i}\cos(\phi_{i})+\psi_{i}$$

where R_i and f_i control the amplitude and frequency of the flapping, respectively, and Δϕ_ij determines the phase lag between adjacent joints, thereby forming a traveling wave motion of the pectoral fin at the macroscopic level. Recent research focus has shifted toward designing topological networks to replicate the “Mobula” and “Raja” modes of real fish. By adjusting the spanwise phase difference parameter Δϕ_span, the controller can smoothly switch between the high-efficiency “flapping mode” (Δϕ ≈ 0, lift-dominated) and the high-maneuverability “undulating mode” (Δϕ ≫ 0, thrust-dominated). This parameter-space-based mode switching mechanism avoids the discrete jumps typical of traditional finite state machines (FSM) during mode transitions, ensuring smooth operation of the actuators. In a review, Wang summarized that the phase lag parameter is critical in determining the swimming mode: when Δϕ ≈ 0, the pectoral fins exhibit a synchronous up-and-down “flapping mode,” where the effective angle of attack is maximized and lift generation efficiency is optimal; when Δϕ > 0, the fins exhibit a flexible wave-like “undulating mode,” providing better thrust continuity suitable for high-maneuverability scenarios [85]. This parameter-space-based switching mechanism [160], ensures the stable operation of the actuators in unsteady flow fields [161].

#### 5.2.2. Kinematic Mapping and Zero-Radius Turn Strategy

While the CPG network outputs reference trajectories in the joint space, the mapping of these low-level signals into task-space maneuvers constitutes a critical link in the control system. One of the most notable maneuverability features of the biomimetic manta ray robot is its ability to execute zero-radius turns. Research by Zhang demonstrated the potential for high-maneuverability swimming through coordinated control of multi-joint structures; in manta ray robots, this capability is primarily achieved through asymmetric flapping of the left and right pectoral fins [162]. Hao conducted in-depth studies on the CPG-based heading control mechanism, quantifying the specific impacts of “amplitude difference” and “phase difference” on turning performance [99]. The study found that for large-radius cruise turns, an amplitude deviation strategy—increasing the amplitude of the outer fin while decreasing that of the inner fin—produces smooth differential thrust. Conversely, for tactical maneuvers in narrow deep-sea terrains such as hydrothermal vent fields, the system must switch to an anti-phase flapping strategy [163]. This strategy, by changing the CPG center offset φi or directly reversing the wave propagation direction, causes the two pectoral fins to execute forward-stroking and backward-stroking motions, respectively, utilizing the difference in fluid form drag to achieve zero-radius turns with high angular velocity. Meng further pointed out that this maneuverability depends not only on control algorithms but also heavily on the flexible structural design of the pectoral fins; optimized flexibility distribution can significantly enhance fluid attachment effects during asymmetric flapping, thereby improving the generation efficiency of turning moments [164].

### 5.3. Dynamic Control and Disturbance Rejection

Open-loop control relying solely on CPGs cannot handle complex deep-sea current disturbances or drifts in model parameters. To achieve tasks such as constant-depth cruising and precise station keeping, closed-loop feedback control based on dynamic models must be introduced. However, since fluid-structure interaction effects are difficult to model precisely, robust control has become the mainstream choice in this field [165].

#### 5.3.1. Robust Control Based on Simplified Models

Although the complete Navier–Stokes equations cannot be used for real-time control, the rigid-body dynamics equations of the biomimetic manta ray can be established by simplifying fluid forces (e.g., using quasi-steady blade element theory) [166]. However, traditional PID controllers often perform poorly when dealing with strong underwater nonlinear drag terms, being prone to integral saturation or overshoot. To address this, He proposed a depth and heading control method based on the S-plane control model [93,167]. The core of the S-plane controller lies in utilizing the nonlinear [92] saturation characteristic of the Sigmoid function to construct the control law, in the form:

(4) $$u=-k_{1}e-k_{2}\dot{e}+f(e,\dot{e})$$

Compared with linear PID, the Sigmoid function provides saturated control moments when the error is large, preventing actuator overload; when the error is small, it exhibits linear regulation to ensure steady-state accuracy. Experiments proved that this method significantly outperforms traditional linear controllers in suppressing overshoot and accelerating convergence, showing extreme robustness particularly when facing unknown hydrodynamic disturbances [165]. Additionally, to address actuator dead zones and input nonlinearities, Cui designed an adaptive sliding mode control [168]. This method effectively mitigates the “chattering” phenomenon inherent in sliding mode control by introducing an adaptive law to estimate the upper bound of external disturbances online, thereby protecting the mechanical transmission of the flexible pectoral fins. To further enhance control intelligence, Cao proposed a strategy combining CPG with fuzzy logic, utilizing fuzzy rules [169] to dynamically adjust CPG frequency and amplitude parameters based on attitude errors, endowing the robot with biomimetic reflex-like disturbance rejection capabilities [88].

#### 5.3.2. Integrated Gliding-Flapping Hybrid Propulsion and Longitudinal Stability Control

In deep-sea long-endurance missions, a single flapping-wing propulsion mode often struggles to meet stringent energy optimization requirements. To address this bottleneck, Zhang, in a paper published in J. Mar. Sci. Eng., innovatively proposed a dual-mode biomimetic manta ray robot design that integrates “gliding” and “flapping” [74]. The core of this architecture is the establishment of a synergistic scheduling mechanism between the VBS and pectoral fin flapping: during the long-distance cruising phase, the robot adjusts its net buoyancy via the VBS and its pitch angle via a center-of-gravity adjustment mechanism to glide with low energy consumption, simulating the motion mechanism of an underwater glider; when facing requirements for rapid maneuverability or station keeping, the system can seamlessly switch to the pectoral fin flapping mode to output high-burst thrust and moments.

In his monograph, Yu further elaborated on the design theory of such systems, noting that for flat-bodied submersibles, longitudinal stability becomes the key constraint on gliding efficiency due to the lack of aerodynamic damping provided by a tail [170]. Advanced control strategies tend to adopt a cascaded control architecture: the inner loop utilizes high-frequency IMU data for rapid attitude stabilization, while the outer loop achieves closed-loop depth control based on depth gauge feedback. Experimental data indicate that this hybrid propulsion strategy, characterized by deep hardware-software synergy, significantly extends the robot’s endurance while ensuring maneuverability, providing a feasible engineering paradigm for kilometer-class deep-sea exploration.

### 5.4. Advanced Intelligent Control: Reinforcement Learning and Policy Transfer

As task complexity increases, the traditional “modeling + control” paradigm reveals its limitations in extreme unstructured environments. Tong pointed out in a review of reinforcement learning (RL) for biomimetic underwater robots [171], hat the analytical models relied upon by traditional methods are often oversimplified and fail to capture the complex dynamic features of fluid-flexible body interaction. In contrast, DRL [172,173], as a data-driven method, provides a new paradigm for resolving high-dimensional, nonlinear biomimetic control problems.

Current intelligent control research primarily utilizes DRL for high-level decision-making (e.g., path planning, gait selection), while the low-level execution still relies on CPG or PID. This approach leverages the stability of traditional control but is limited by the performance ceiling of the low-level controllers.

To break through this limitation, recent studies have begun to explore end-to-end learning paradigms. Work published by Lin in IEEE Robotics and Automation Letters demonstrated an end-to-end reinforcement learning method that completely abandons CPGs [174]. By directly mapping the robot’s state to joint actuator commands without a preset periodic signal generator, the neural network not only autonomously evolved highly efficient bio-like swimming gaits but also demonstrated non-periodic agile maneuverability (e.g., rapid stops, instantaneous large-angle turns) that is difficult to achieve with traditional CPGs. This indicates that by shedding the constraints of biomimetic prior knowledge, neural networks may discover superior control manifolds.

Although simulation results are encouraging, the Sim-to-Real gap remains the greatest obstacle to the implementation of reinforcement learning. Tong emphasized that due to the high computational cost of underwater hydrodynamic simulation, existing simulation environments often adopt simplified fluid models, causing policies to fail in real environments [27,171,175,176]. To resolve this, Hasan suggested introducing domain randomization and meta-learning techniques to enhance policy robustness by incorporating uncertainties from flow field disturbances and sensor noise during training [153,177]. Future research will focus on constructing high-fidelity, low-latency simulation platforms and developing adaptive reinforcement learning algorithms with online adaptation capabilities, truly achieving embodied intelligence for biomimetic manta ray robots in deep-sea environments.

### 5.5. Chapter Summary

Based on the engineering application requirements in deep-sea environments, this chapter systematically constructs a complete framework for translating biological mechanisms into engineering control logic for biomimetic manta ray robots. Addressing the challenges of unsteady fluid-structure interaction and strong nonlinear dynamics generated by high-aspect-ratio pectoral fins, this chapter first establishes a kinematic dimension reduction model via CPGs. Utilizing a bilaterally symmetrical chain topology, it replicates the rhythmic undulations of biological pectoral fins and achieves seamless switching between “flapping” and “undulating” modes through dynamic adjustment of phase lag parameters, providing fundamental motion primitives for complex maneuvers and zero-radius turns.

At the dynamic level, addressing disturbances in unstructured deep-sea environments, this chapter introduces S-plane control with nonlinear saturation characteristics and adaptive sliding mode strategies, significantly enhancing the robustness of the closed-loop system. Combined with the variable buoyancy system, a “gliding-flapping integrated” hybrid propulsion scheme is proposed, effectively balancing the economy of long-endurance cruising with the high efficiency of instantaneous maneuverability. Finally, this chapter explores the potential of deep reinforcement learning in resolving high-dimensional nonlinear mappings and end-to-end control, analyzing key technologies to overcome the Sim-to-Real gap. This establishes the theoretical and engineering foundation for achieving embodied intelligence and autonomous evolution of biomimetic manta ray robots in extreme deep-sea environments.

## 6. Summary and Outlook

### 6.1. Overall Summary

From the perspective of multi-physical field coupling and system evolution, this paper has comprehensively explored the taxonomy, structural paradigms, energy strategies, and motion control of biomimetic manta ray submersibles governed by operational depth. By systematically surveying global research lineages, this review reveals how operational depth, as a core physical parameter, dictates the profound evolution of design paradigms. During the shallow-water experimental stage, research centered on the precise replication of biological unsteady hydrodynamic mechanisms and resolving the mechanical simulation of complex flapping motions. As the operational environment extended into mid-to-deep sea regions, the core contradiction shifted toward system integration, depth-keeping stability, and energy efficiency management through “gliding-flapping” integration. Finally, in extreme full-ocean-depth environments, the design philosophy achieved a disruptive leap from “resisting pressure” to “adapting to pressure,” establishing the technical paradigm of pressure equalization.

Research further indicates that “rigid-flexible integration” represents the optimal path for resolving the mechanical paradox between deep-sea high pressure and flexible propulsion. Purely rigid structures often fall into energy-efficiency traps, while purely soft structures face challenges regarding insufficient force output and control nonlinearity. By mimicking the gradient distribution characteristics of biological manta rays—characterized by an “internally rigid and externally flexible, proximally hard and distally soft” construction—the rigid skeleton serves as a power transmission medium to maintain the hydrodynamic shape, while the flexible skin induces a stable LEV through passive deformation. This synergy significantly enhances both propulsive efficiency and maneuverability.

In the domains of energy and control, systems are undergoing a deep collaborative evolution toward biologization and intelligence. To circumvent the weight penalty associated with traditional pressure hulls, energy systems are transitioning toward structural-functional integration. PBOF technology and “fuselage-is-battery” structural battery schemes have become key to enabling long-endurance operations. Simultaneously, control architectures have progressed from traditional paradigms dependent on precise analytical models toward CPG control inspired by biological spinal cords and DRL with autonomous evolutionary capabilities. This intelligent evolution endows robots with “embodied intelligence” to perceive complex vortex fields and actively adapt to unstructured environments, gradually narrowing the performance gap between biological prototypes and engineering equipment.

### 6.2. Future Outlook

Looking forward, the developmental logic of deep-sea biomimetic manta ray robots is undergoing a profound transformation. Research is no longer limited to the enhancement of single-dimensional performance metrics but is moving steadily into “deep water” areas such as reliability design, standardization of manufacturing processes, and systematization of operations. This engineering evolution will reshape the form and capability of deep-sea exploration equipment through a systematic reconfiguration across four dimensions: structure, energy, synergy, and application.

First, in the dimension of structural manufacturing, the body of biomimetic robots will achieve a fundamental evolution from “assembly integration” toward “voxel-level gradient manufacturing”. Addressing the risks of existing rigid-flexible coupled structures—which rely heavily on mechanical joints and have physical interfaces prone to failure under high pressure—future manufacturing processes will abandon traditional assembly logic in favor of advanced multi-material 3D or even 4D printing technologies. These technologies enable a continuous gradient transition from rigid skeletons to flexible skins at the voxel level, thereby completely eliminating macroscopic interfaces at the physical level and resolving delamination issues under cyclic deep-sea loads. Furthermore, by incorporating smart materials such as electroactive polymers, these integrated structures will endow robots with “fluid perception” capabilities to adjust their modulus in real time, making them mechanically more similar to real organisms [178].

Second, energy systems will witness a revolutionary breakthrough from “passive carrying” toward “in-situ metabolism”. To break the endurance constraints of deep-sea operations, future designs will focus on constructing a “residency-replenishment” machine metabolic system. This involves deepening structural battery technology to embed solid-state energy storage units directly into the composite materials of the body—achieving a “fuselage-is-energy” configuration that significantly reduces parasitic mass. Additionally, in coordination with deep-sea infrastructure, research will aggressively develop high-power underwater wireless charging and environmental energy harvesting (e.g., ocean thermal energy conversion OTEC and triboelectric nanogenerators TENG). This transformation will endow robots with a bio-like “hibernation-feeding (charging)” cycle, theoretically enabling infinite residency in deep-sea environments.

Furthermore, the operational mode will transition from single-unit execution toward the construction of an ecosystem based on heterogeneous swarm synergy [179,180,181,182]. Driven by decentralized control architectures powered by DRL, future biomimetic schools will be heterogeneously composed of “mother-units” (responsible for long-endurance relay) and “daughter-units” (responsible for high-maneuverability detection). By utilizing underwater biomimetic communication mechanisms such as acoustic field modulation or vortex signals, these multi-agent systems will achieve efficient task allocation and collaborative mapping. This will culminate in a distributed detection “ecosystem” covering the wide-area deep sea, greatly enhancing operational efficiency and robustness [183].

Ultimately, we expect biomimetic manta ray robots to transition from laboratory prototypes to all-domain adaptive operations. Focus will be placed on three major engineering scenarios: first, constructing cross-medium penetration platforms that break the physical boundaries of air-water interfaces to achieve seamless switching and rapid deployment across air, water, and seafloor domains; second, developing deep-sea in situ scientific nodes that serve as mobile ocean observation stations integrating multi-physical field sensors for long-term environmental monitoring and ecological surveys; and third, serving strategic defense and reconnaissance by leveraging low-noise, stealthy biological characteristics for close-in reconnaissance of high-value targets and maintenance of deep-sea facilities in sensitive waters.

## Figures and Tables

**Table 2:** Key technical parameters of NPU biomimetic manta ray submersible prototypes [28].

Model No.	Year	Wing Structure	Dimensions/Weight	Performance (Speed/Depth)	Endurance	Typical Payload
**I**	2019	Fully Flexible	Wingspan 0.8 m, 10 kg	0.5–1 km, shallow-water test	5–8 h	Stereo camera, high-performance industrial PC, laser rangefinder
**II**	2019	Fully Rigid	Wingspan 2.0 m, 120 kg	0.5–1 km, depth 500 m	30-day glide, 1000 km	Altimeter, CTD (25 kg payload)
**III**	2020	Fully Flexible	Wingspan 2.0 m, 100 kg	1–2 km, depth 500 m	30-day glide, 500 km	Stereo camera, altimeter, CTD (20 kg payload)
**IV**	2021	Semi-Flexible [100]	Wingspan 3.0 m, 500 kg	1–2 km, depth 1000 m	30-day glide, 1000 km	Stereo camera, altimeter, CTD (20 kg payload)
**V**	2022	Fully Flexible	Wingspan 1.2 m, 30 kg	1–2 km, shallow-water maneuver	5~8 h	Vector hydrophone, DVL, water quality monitor
**VI**	2023	Fully Flexible	Wingspan 4.2 m, 800 kg	2–3 km, depth 1000 m	30-day glide, 1000 km	High-performance control system, optical/acoustic/magnetic multi-source sensors (50 kg payload)

**Table 3:** Detailed technical specification comparison between Sea Guru I and Sea Guru II [102].

	Sea Guru I	Sea Guru II
Dry Weight (Air)	741.281 kg	930 kg
System Voltage	330VDC, 24VDC, 12VDC;	330VDC, 24VDC, 12VDC
Propulsion	Bionic Side Fins, Main Thrusters: 2,Vertical Thrusters: 3	Bionic Undulating Fin, Main Thrusters: 2, Vertical Thrusters: 4
Energy	LiFePO4 Battery, Built-in BMS, Main Battery Capacity: 3 kW·h, 10 Ah, Cell Count: 86	LiFePO4 Battery, Built-in BMS, Main Battery Capacity: 3 kW·h, 10 Ah, Cell Count: 86
Observation and Communication	HD Camera, at least single-channel with local storage > 2 h; Sensors: AHRS, T/D Sensor, Acoustic Beacon, Leak Sensor	HD Camera, at least dual-channel with local storage > 2 h; Sensors: AHRS, T/D Sensor, Acoustic Beacon, Leak Sensor, DVL, CTD, DO, pH
Communication	Microfiber Optic, UHF	Microfiber Optic, UHF
Control	Manual Operation, Automatic Altitude Hold, Attitude Adjustment Manual Operation	Manual Operation, Attitude Adjustment, Path Navigation
Relay Station	Capable of 2000 m Deployment	Capable of 2000 m Deployment
Wingspan	Current Wingspan 3.4 m (Foldable to 2.2 m for Deployment)	Current Wingspan 3.1 m
Body and Tail Length	Current Length 3 m	Current Length 4 m
Body Height	Current Height 0.7 m	Current Height 0.75 m
Single Undulating Fin Width	Current Fin Width 0.98 m	Current Single Fin Width 0.7 m

## Data Availability

No new data were created or analyzed in this study..
